# Pharmacokinetic/pharmacodynamic evaluation of gamithromycin against rabbit pasteurellosis

**DOI:** 10.1186/s12917-024-03988-y

**Published:** 2024-04-20

**Authors:** Xin-Yi Wei, Jing Zhang, Yin Zhang, Wen-Zhen Fu, Long-Gen Zhong, Yi-Duo Pan, Jian Sun, Xiao-Ping Liao, Ya-Hong Liu, Yu-Feng Zhou

**Affiliations:** 1 Guangdong Laboratory for Lingnan Modern Agriculture, National Risk Assessment Laboratory for Antimicrobial Resistance of Animal Original Bacteria, College of Veterinary Medicine, South China Agricultural University, Guangzhou, China; 2 Guangdong Provincial Key Laboratory of Veterinary Pharmaceutics Development and Safety Evaluation, South China Agricultural University, Guangzhou, China; 3 Yantai Fushan Center for Animal Disease Control and Prevention, Fushan, Yantai, Shandong China

**Keywords:** PK/PD, Gamithromycin, *P. Multocida*, Rabbit

## Abstract

**Background:**

Gamithromycin is an effective therapy for bovine and swine respiratory diseases but not utilized for rabbits. Given its potent activity against respiratory pathogens, we sought to determine the pharmacokinetic profiles, antimicrobial activity and target pharmacokinetic/pharmacodynamic (PK/PD) exposures associated with therapeutic effect of gamithromycin against *Pasteurella multocida* in rabbits.

**Results:**

Gamithromycin showed favorable PK properties in rabbits, including high subcutaneous bioavailability (86.7 ± 10.7%) and low plasma protein binding (18.5–31.9%). PK analysis identified a mean plasma peak concentration (C_max_) of 1.64 ± 0.86 mg/L and terminal half-life (T_1/2_) of 31.5 ± 5.74 h after subcutaneous injection. For *P. multocida*, short post-antibiotic effects (PAE) (1.1–5.3 h) and post-antibiotic sub-inhibitory concentration effects (PA-SME) (6.6–9.1 h) were observed after exposure to gamithromycin at 1 to 4× minimal inhibitory concentration (MIC). Gamithromycin demonstrated concentration-dependent bactericidal activity and the PK/PD index area under the concentration-time curve over 24 h (AUC_24h_)/MIC correlated well with efficacy (R^2^ > 0.99). The plasma AUC_24h_/MIC ratios of gamithromycin associated with the bacteriostatic, bactericidal and bacterial eradication against *P. multocida* were 15.4, 24.9 and 27.8 h in rabbits, respectively.

**Conclusions:**

Subcutaneous administration of 6 mg/kg gamithromycin reached therapeutic concentrations in rabbit plasma against *P. multocida*. The PK/PD ratios determined herein in combination with ex vivo activity and favorable rabbit PK indicate that gamithromycin may be used for the treatment of rabbit pasteurellosis.

**Supplementary Information:**

The online version contains supplementary material available at 10.1186/s12917-024-03988-y.

## Background

*Pasteurella multocida* and rabbit hemorrhagic disease virus infections are considered the most devastating diseases in rabbit industry due to their major economic losses [1]. In particular, *P. multocida* is usually associated with coinfections by other bacteria such as *Bordetella bronchiseptica* and *Staphylococcus aureus* [2]. Previous studies showed a mortality rate of > 50% in a grower rabbit population that was diagnosed with mixed bacterial infections due to *P. multocida* and *S. aureus* [3, 4]. Based on the capsular antigens, *P. multocida* can be grouped into five serogroups (A to F) which are further classified into 16 somatic serotypes based on the composition of lipopolysaccharide antigens [5, 6]. The *P. multocida* serogroups A, B and F are the primary serotypes responsible for rabbit pasteurellosis [7, 8]. In addition to typical respiratory symptoms (snuffles to pneumonia), rabbit pasteurellosis is often accompanied by the occurrence of otitis media, conjunctivitis, abscesses, genital infections, and septicemia [7, 9]. At present, vaccination is an alternative therapeutic approach to prevent pasteurellosis. However, currently available vaccines do not provide life-long and complete cross protections due to heterogeneity among multiple *P. multocida* serotypes [6]. Thus, antibiotic therapy is still an important control method for rabbit pasteurellosis [10].

Gamithromycin is a semi-synthetic macrolide antibiotic approved for treating bovine and swine respiratory diseases caused by *P. multocida*, *Haemophilus parasuis*, *Actinobacillus pleuropneumoniae* and *Mannheimia haemolytica* [11, 12]. Previous studies in cattle, swine and turkey have demonstrated considerable antimicrobial efficacies of gamithromycin therapy against *Streptococcus suis* and *Ornithobacterium rhinotracheale* [13–15]. Based on our previous pharmacokinetic/pharmacodynamic (PK/PD) studies about gamithromycin, ex vivo AUC_24h_/MIC ratio was chosen as the predictive pharmacodynamic index associated with ex vivo activity of gamithromycin against *H. parasuis* and *S. suis* [15, 16]. However, gamithromycin PK/PD relationship for rabbit pasteurellosis still remained unclear. Considering the wide-spectrum activities of gamithromycin against these respiratory pathogens, we thus investigated ex vivo activity and PK/PD correlation of gamithromycin against *P. multocida*.

Although gamithromycin PK studies have been previously reported in many species of animals including cattle, swine, foal and broiler chicken [16–19], there are limited PK observational data in rabbit. Such PK data are the prerequisite in the PK/PD modeling analysis for gamithromycin. We therefore aimed to establish a PK/PD approach to evaluate the precise concentration-response relationship of gamithromycin against *P. multocida* in rabbits. In addition, this study described the PK profiles and plasma protein binding of gamithromycin in rabbits, and determined the post-antibiotic effect (PAE) and post-antibiotic sub-inhibitory concentration effect (PA-SME) of gamithromycin against *P. multocida*. Specifically, the magnitudes of PK/PD parameter AUC_24h_/MIC ratios required for efficacy against *P. multocida* were examined in rabbits to provide a framework for further development of drug-dosing regimens to optimize gamithromycin therapy against rabbit pasteurellosis.

## Results

### In vitro susceptibility testing and time-killing curves

The minimal inhibitory (MIC) and bactericidal (MBC) concentrations of gamithromycin against *P. multocida* CVCC 434 in cation-adjusted Mueller-Hinton (CAMH) broth were 0.125 and 0.25 mg/L, respectively. Interestingly, the MIC of gamithromycin for this strain was determined to be 0.03125 mg/L in rabbit plasma, which was four-fold lower than the MIC value in CAMH broth. This hinted at a trend of higher improvement in antibacterial potential of gamithromycin in plasma.

**Fig. 1:**
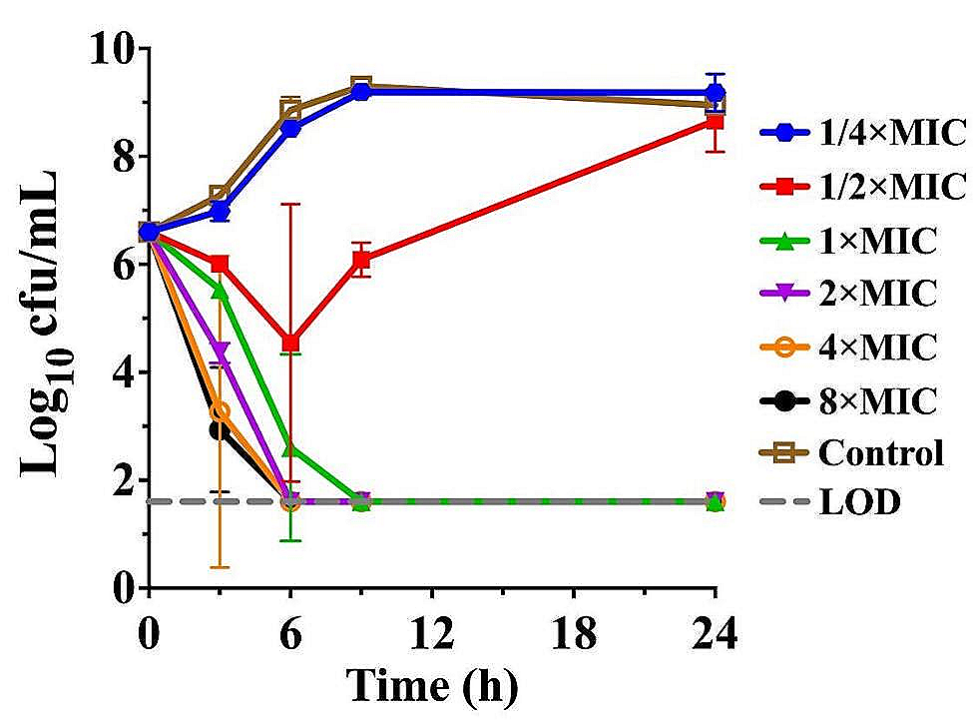
In vitro time-killing curves of gamithromycin against *P. multocida* CVCC 434 in CAMH broth. The limit of detection (LOD) was 40 cfu/mL

Rapid killing was demonstrated with gamithromycin at a sub-MIC concentration of 0.063 mg/L against *P. multocida*, with a > 2.0 log_10_ cfu/mL reduction after 6 h, followed by regrowth whereby bacterial density mimicked that of the control within 24 h (Fig. 1). A concentration-dependent trend towards a greater level of killing activity was observed in the presence of 1 to 8× MICs gamithromycin. Complete bactericidal activity reaching the undetectable limits of eradication was achieved within 6 h in response to gamithromycin at 2×MIC or higher concentrations (Fig. 1).

### PAE and PA-SME determinations

**Fig. 2:**
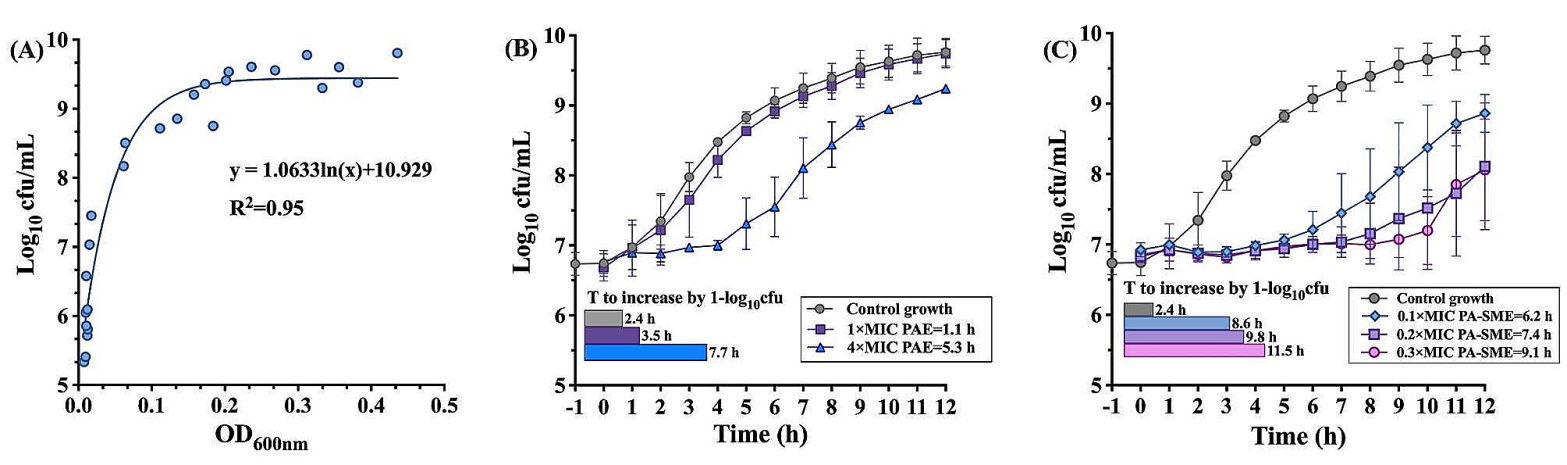
In vitro PAE and PA-SME values for gamithromycin against *P. multocida*CVCC 434. (**A**) Standard curve constructed by regression of the viable bacterial counts and absorbance (OD_600nm_) of *P. multocida* CVCC 434. The solid points represent the observed data and the line represents the best fitting curves as follows: y = 1.0633 ln(x) + 10.929 (R^2^ = 0.9453). (**B-C**) The PAEs (**B**) were measured after initial exposure to gamithromycin at 1× and 4× MIC against *P. multocida* CVCC 434; The PA-SME (**C**) was determined after an initial exposure to gamithromycin at 4×MIC. The horizontal bars represent the time required for viable counts of bacteria to increase by 1.0 log_10_ cfu/mL in the drug removal (PAE) and sub-MIC phase (PA-SME)

During the measurements of gamithromycin PAE and PA-SME for *P. multocida*, the regression curve equation of Y = 1.0633 ln(X) + 10.929 (**Fig. 2A**; *R*^2^ = 0.95) was used for converting the absorbance (OD_600nm_) into bacterial counts. Removal of gamithromycin after drug exposure for 1 h resulted in delayed regrowth (PAE) that ranged from 1.1 to 5.3 h (Fig. 2B). A higher level of exposure to gamithromycin resulted in a longer in vitro PAE. After the initial exposure to gamithromycin at 4× MIC and then spiking with sub-MIC levels of gamithromycin at 0.1 to 0.3×MIC, a further regrowth delay was observed, generating PA-SMEs ranged from 6.2 to 9.1 h (Fig. 2C).

### PK profiles and plasma protein binding of gamithromycin in rabbits

**Fig. 3:**
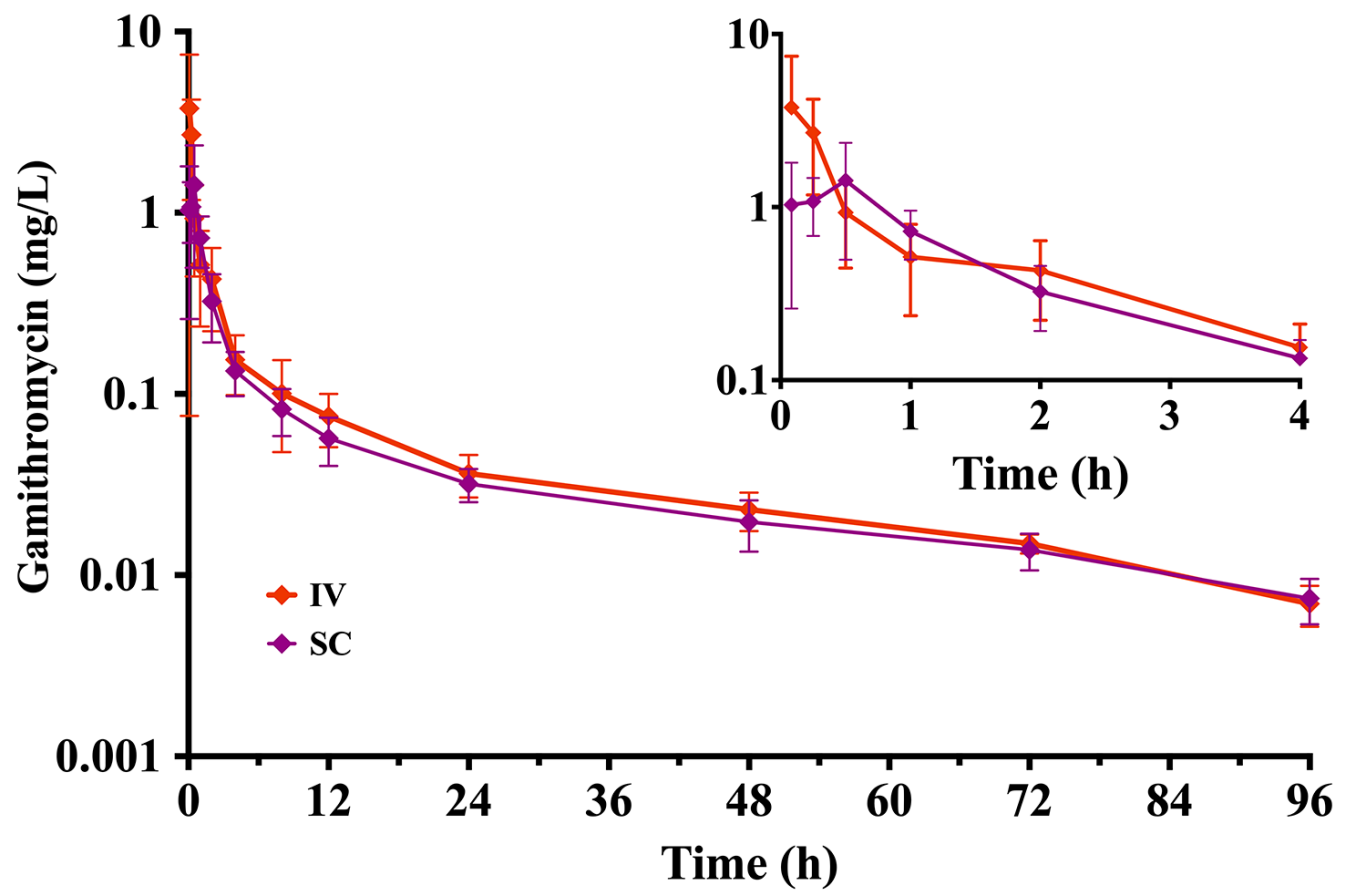
The plasma time-concentration profiles of gamithromycin in New Zealand rabbits received a single dose intravenous (IV) or subcutaneous (SC) administration at 6 mg/kg (*n* = 9 per time point). Upper right panel shows details from 0 to 4 h

No animal experienced apparent adverse effects from the treatment at any time point, and all animals have completed the study. The mean (± SD) concentration-time curves of gamithromycin in rabbit plasma following single-dose intravenous (IV) and subcutaneous (SC) administrations at 6 mg/kg were represented in Fig. 3, and their non-compartmental pharmacokinetic parameters were summarized in Table 1. Overall, gamithromycin exhibited a prolonged terminal half-life (T_1/2λz_, 31.5 ± 5.74 h) and high subcutaneous bioavailability (F%, 86.7 ± 10.7%; Table 1). The protein binding percentages of gamithromycin in rabbit plasma were shown in Table 2, indicating a mean percent protein binding of 25.7% in the concentration range of 0.1 to 10 mg/L.

**Table 1:** Pharmacokinetic parameters (mean ± SD, *n* = 9) of gamithromycin in New Zealand rabbits after single intravenous (IV) and subcutaneous (SC) administrations at 6 mg/kg

PK parameters	Unit	IV	SC
λz	1/h	0.03 ± 0.01	0.02 ± 0.01
T_1/2λz_	h	24.5 ± 6.23	31.5 ± 5.74
C_max_	mg/L	NA	1.64 ± 0.86
T_max_	h	NA	0.37 ± 0.26
AUC_0 − 24 h_	mg·h/L	3.94 ± 0.56	3.25 ± 0.37
AUC_0 − t_	mg·h/L	5.26 ± 1.05	4.37 ± 0.67
AUC_0−∞_	mg·h/L	5.66 ± 0.98	4.91 ± 0.60
MRT_last_	h	22.3 ± 6.18	27.2 ± 7.35
V_z_	L/kg	38.8 ± 12.8	57.0 ± 13.2
Cl	L/kg/h	1.07 ± 0.15	NA
Cl/F	L/kg/h	NA	1.24 ± 0.15
F	%	NA	86.7 ± 10.7

λ_z_, terminal elimination rate; T_1/2λz_, terminal half-life; C_max_, peak concentration; AUC, area under the concentration-time curve from 0 to 24 h (AUC_0 − 24 h_), from 0 to the last sampling point (AUC_0 − t_), or from 0 to ∞ (AUC_0−∞_); MRT_last_, mean residence time; V_z_, volume of distribution; Cl, body clearance; Cl/F, clearance scaled by bioavailability; F, bioavailability; NA, not applicable

**Table 2:** Percent protein binding of gamithromycin in rabbit plasma at the spiked drug concentrations of 0.1, 1 and 10 mg/L

Spiked gamithromycin levels (mg/L)	Mean percent binding (%; ± SD, *n* = 3)
0.1	31.9 ± 0.54
1	26.6 ± 1.24
10	18.5 ± 1.30

### Ex vivo** antimicrobial activity and PK/PD analysis**

**Fig. 4:**
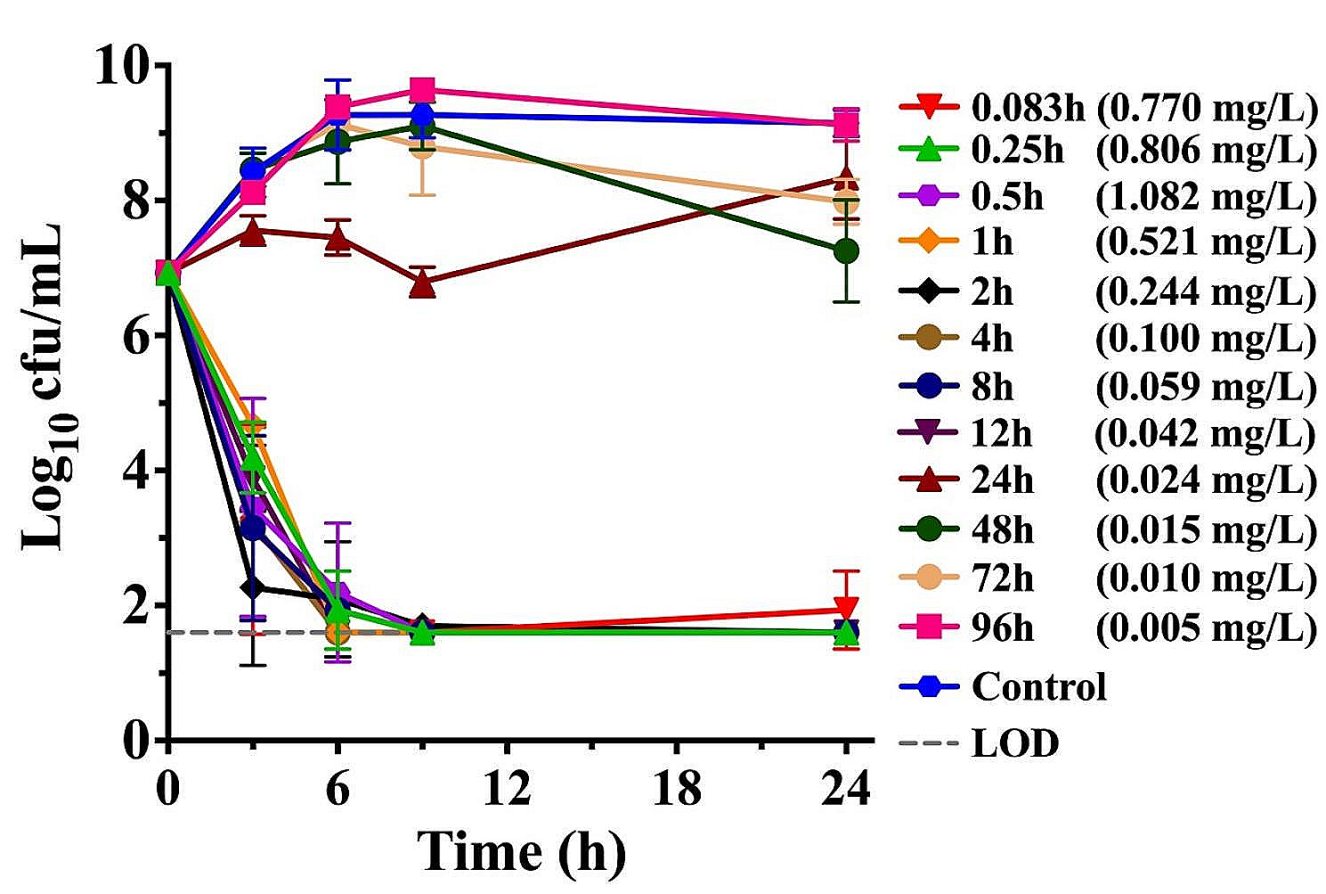
Ex vivo activity of gamithromycin for *P. multocida* CVCC 434 in plasma samples from the rabbits receiving subcutaneous administration of gamithromycin at 6.0 mg/kg. Numerical values on the right brackets represent the mean free drug concentrations of gamithromycin in rabbit plasma collected at different time points

Consistent with the results of in vitro time-killing experiments performed in CAMH broth, the concentration-dependent ex vivo antimicrobial activity was observed for *P. multocida* CVCC 434 in rabbit plasma. Sustained killing activities were achieved for rabbit plasma collected up to 12 h with free drug concentrations of 0.042 to 1.082 mg/L after SC administration of gamithromycin at 6.0 mg/kg (Fig. 4). Bacterial counts of *P. multocida* were below the detectable limits in the plasma collected up to 4 h at 9 h of incubation. Ex vivo killing activity was negligible in plasma at 72 h and 96 h, while bacterial growth inhibition activity was noted within 9 h of exposure to rabbit plasma at 24 h and was followed by moderate regrowth by 24 h of incubation (Fig. 4). In fact, plasma collected from rabbits at 24 h contained a mean free gamithromycin level of 0.024 mg/L that was slightly lower than the MIC for *P. multocida* CVCC 434 in plasma (0.03125 mg/L). This may be responsible for the bacterial regrowth after exposure to 24 h plasma samples.

**Fig. 5:**
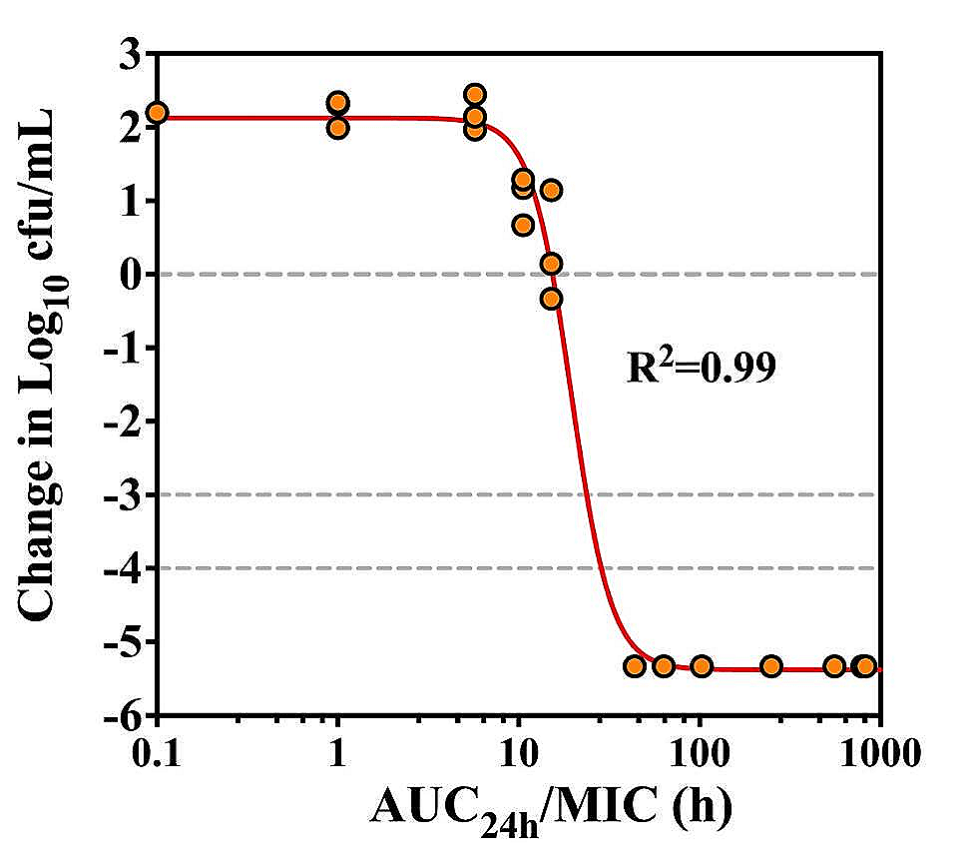
PK/PD relationship of gamithromycin against *P. multocida*. Correlation plot of ex vivo antimicrobial activity and AUC_24h_/MIC ratio of gamithromycin based on the sigmoid E_max_ equation. The line represents the fitting curve of the predicted values, and the points represent observed values of plasma samples collected at time points from 0 to 96 h

The relationship between the PK/PD index AUC_24h_/MIC and ex vivo activity was analyzed using the Sigmoid E_max_ model. Similar to other macrolides [16, 20, 21], the AUC_24h_/MIC ratio was a robust predictor of antimicrobial activity for gamithromycin against *P. multocida*, with an R^2^ of > 0.99 (Fig. 5). The greatest bactericidal effect with a 5.37 log_10_ cfu/mL bacterial reduction was achieved in rabbit plasma after SC administration of gamithromycin at 6.0 mg/kg. Against *P. multocida*, the AUC_24h_/MIC ratios for gamithromycin required to achieve the bacteriostatic, bactericidal and bacterial eradication in rabbit plasma were 15.4, 24.9 and 27.8 h, respectively (Table 3). This implies that gamithromycin was capable of achieving a net bacteriostatic effect for *P. multocida* when the plasma concentrations reached the 0.64×MIC.

**Table 3:** PK/PD analysis and AUC_24h_/MIC ratio values of gamithromycin necessary to achieve the bacteriostatic, bactericidal and eradication effects against *P. multocida* CVCC 434 in rabbit plasma

PK/PD parameters ^a^	Units	Mean ± SD ^b^
E_0_	log_10_ cfu/mL	2.42 ± 0.38
E_max_	log_10_ cfu/mL	-5.37 ± 0.01
E_max_ - E_0_	log_10_ cfu/mL	-7.51 ± 0.11
EC_50_	h	19.5 ± 1.59
N	NA	4.12 ± 0.67
AUC_24h_/MIC for bacteriostatic	h	15.4 ± 1.79
AUC_24h_/MIC for bactericidal	h	24.9 ± 2.25
AUC_24h_/MIC for eradication	h	27.8 ± 0.52

^*a*^ E_0_, the change in bacterial count after 24 h of incubation in control plasma; EC_50_, the AUC_24h_/MIC ratio associated with 50% of the maximal activity (E_max_); N, the slope of the PK/PD relationship profile; the bacteriostasis, bactericidal and eradication were defined as the net static, 3-log_10_, and 4-log_10_ kill effects after 24 h of incubation; NA, not applicable. ^*b*^ Data were acquired from three independent evaluations for ex vivo activity of gamithromycin in rabbit plasma

## Discussion

To our knowledge, this is the first report of gamithromycin PK characteristics in rabbits, while its PK evaluations have already been reported in other species of animals [19, 20, 22, 23]. After subcutaneous injection of gamithromycin, the plasma drug concentration reached a maximum of 1.64 mg/L in rabbits, which was nearly as twice as that of C_max_ (0.75 mg/L) obtained in cattle [24]. The T_max_ in plasma (0.37 h) tended to be shorter for gamithromycin in rabbits compared to that in cattle (1 h), sheep (0.91 h), and pigs (0.63 h) [12, 24, 25], indicating that gamithromycin was absorbed rapidly into the systemic circulation. More importantly, gamithromycin has the great advantage of being potentially administered as a single dose for treating bacterial infections in rabbits due to its long terminal half-life (31.5 ± 5.74 h) identified in this study. In addition, an even slower elimination profile of gamithromycin was observed in cattle [24], with a more prolonged terminal half-life (50.8 h) compared to rabbit. Of note, gamithromycin exhibited a very high volume of distribution (V_z_, 57.0 ± 13.2 L/kg) in rabbits after SC administration, which suggested excellent tissue distribution.

Macrolides are frequently used as antibiotics to treat respiratory infections due to their strong tissue penetrations into lung, resulting in lung concentrations that often exceed the plasma concentrations [26, 27]. Previous PK studies in other species such as cattle and foal (not rabbit) demonstrated that as a veterinary-specific macrolide antibiotic, gamithromycin was able to achieve a 4.7 to 127 fold accumulation in the pulmonary epithelial lining fluid (PELF) compared to in plasma after administration of a single SC or intramuscular dose at 6 mg/kg [17, 23]. This is further supported by another previous study in cattle, where the lung AUC_0−∞_ value of gamithromycin was 194 times higher than the corresponding plasma AUC_0−∞_ value [24]. In addition, gamithromycin has been shown to express a longer terminal half-life in cattle lung tissue (93 h) than that in plasma (62 h) [23], which could be more conducive to the treatment of respiratory infections. Whilst bronchopulmonary disposition studies with gamithromycin have not been conducted in rabbits until now, the livestock-derived results of good PELF penetration may suggest good models of penetration into rabbit lung tissue.

Our study demonstrated that the PAEs and PA-SMEs of gamithromycin against *P. multocida* varied in the range of 1.1–5.3 h and 6.3–9.1 h, respectively. This is slightly higher than our previously reported gamithromycin PAEs against *S. suis* (0.5–2.6 h) and *H. parasuis* (1.5–2.4) [15, 16]. However, for the long-acting macrolide antibiotic exhibiting such prolonged half-life in vivo, the clinical significance of these relatively short PAEs may be limited for potential dose regimen adjustments. Typically, with the significantly prolonged terminal half-life in rabbits, effective plasma concentrations of gamithromycin could be maintained for long enough to exceed the MIC value, albeit it was only administered once.

PK/PD assessment of antibiotic efficacy is a critical step in drug development to determine the optimal dose regimen for clinical practice [28]. Previous studies have indicated that the PK/PD index of AUC_24h_/MIC was highly correlated to the efficacy of gamithromycin [29]. We thus used this index in this study for PK/PD attainment measurement as the AUC_24h_/MIC ratio was a robust predictor of antimicrobial activity with an R^2^ of > 0.99 [30]. While this is the first gamithromycin PK/PD evaluation in rabbits, similar PK/PD relationship studies have been previously performed in cattle [29], swine [14], and murine models [20] against *P. multocida*. However, a distinct variation in PK/PD targets of gamithromycin was observed among different animal species, despite all the reported infections caused by *P. multocida*. For example, an AUC_24h_/MIC ratio of 56.77 h was reported to be necessary for gamithromycin to achieve the bacteriostatic action of *P. multocida* in a murine lung infection model [20], which is considerably higher than that of 15.4 h in rabbits here. In light of this, future studies evaluating the in vivo efficacy and PK/PD relationship of gamithromycin in the target animal of rabbit are therefore warranted. In addition, previous evaluations of other macrolide antibiotics such as tulathromycin and tildipirosin in calf and swine demonstrated a similar bacteriostatic AUC_24h_/MIC ratio of 17.28–18.91 h against *P. multocida* [30, 31]. More importantly, a clinical therapy study with gamithromycin in cattle identified a significant association between treatment success and PELF AUC_24h_/MIC ratio of 31 h for *P. multocida* infections [29]. In a multicenter farm trial of bovine respiratory disease associated with *P. multocida*, morbidity in the gamithromycin-treated cattle was reduced by 64% compared with their controls [11].

Rabbit plasma pharmacokinetics in the present study demonstrated that the AUC_0 − 24 h_ value after a single 6 mg/kg SC dose of gamithromycin is 3.25 mg·h/L. Using the most conservative PK/PD estimates identified in this study, a bacteriostatic effect (AUC_24h_/MIC of 15.4 h) would be expected to be achievable for rabbit-derived *P. multocida* isolates with gamithromycin MICs of < 0.211 mg/L. This MIC threshold is somewhat lower than the gamithromycin MIC_90_ value of 1.0 mg/L for bovine *P. multocida* strains [17]. In fact, due to the limited MIC distribution of rabbit-origin *P. multocida* isolates, the current MIC breakpoint in rabbit still remains unclear. Of note, a large potentiation effect of calf serum on the potency of tulathromycin was observed for *P. multocida* [32]. Our previous publications also demonstrated that porcine serum similarly enhanced the antimicrobial activities of gamithromycin and tulathromycin against *H. parasuis* and *S. suis* by increasing intracellular antibiotic uptake and reducing bacterial efflux pump [15, 16, 21]. Together, given the favorable PK profiles of gamithromycin in rabbit with rapid absorption as well as prolonged half-life, low plasma protein binding, increased in vitro potency in plasma, excellent ex vivo activity and PK/PD ratios observed in our study, we thus speculate that gamithromycin has the potential to serve as a promising antibacterial agent for treating rabbit pasteurellosis.

Our investigation has several limitations. For example, we assessed only one standard *P. multocida* strain. Further studies are warranted for a wider range of MIC distribution from rabbit-origin clinical *P. multocida* isolates. In addition, the clinical relevance of this study may be of limited due to the ex vivo PK/PD evaluation. Future studies evaluating the in vivo PK/PD relationship of gamithromycin in rabbits might be even more meaningful. Moreover, we do not know whether gamithromycin could be approved for rabbit pasteurellosis in the future despite the high activity for *P. multocida* infections. Although this is beyond the scope of the present study, gamithromycin would be a promising treatment option for *P. multocida* infections in clinical veterinary practice.

## Conclusion

In summary, this study demonstrated a favorable PK profile of gamithromycin with prolonged half-life in rabbits and exhibited the ex vivo PK/PD relationship against *P. multocida*. Both bacteriostasis and bactericidal effects were achieved herein. The PK/PD ratios determined in this study, along with rabbit PK data and MIC distribution, will be useful in guiding clinical dosing regimen optimization for gamithromycin.

## Methods

### Bacterial strain, media and antibiotics

A well-characterized *P. multocida* CVCC 434 (type B, serotype 2) was obtained from the National Center for Veterinary Culture Collection of China (Beijing, China). The original bacterial culture was isolated from a piglet that died of plague in Jiang Su, China. The strain was maintained, grown, subcultured, and quantified using the CAMH broth and agar (Difco Laboratories, Detroit, MI) with 5% defibrinated sheep blood. Analytical-grade gamithromycin powder was purchased from NMT Biotech (Suzhou, China) and reconstituted in 0.015 mol/L citrate buffer to a final concentration of 1280 mg/L stock solution, with pH adjusted to 7.0. Gamithromycin injection (Zactran, 15% w/v, 150 mg/mL) for the rabbit PK studies was obtained commercially from Boehringer Ingelheim Animal Health (Toulouse, France).

### In vitro susceptibility testing and time-killing curves

The MIC of gamithromycin against *P. multocida* CVCC 434 was determined by the broth microdilution method in accordance with the CLSI guidelines [33]. The *Streptococcus pneumoniae* ATCC 49,619 served as the MIC quality control strain. MIC was defined as the lowest drug concentration of gamithromycin that inhibited the visible bacterial growth in CAMH broth. MBC was determined using the spot-plate technique to obtain a 3-log_10_ reduction in bacterial count compared to the initial inoculum [21]. All MIC and MBC determinations were performed in duplicate with three biological replicates. The median MIC and MBC of replicate assays were reported.

In vitro time-killing experiments of gamithromycin against *P. multocida* CVCC 434 were performed using the CAMH broth with an initial inoculum of ~ 3 × 10^6^ cfu/mL as previously described [21]. Briefly, suspensions of *P. multocida* cells in exponential phase were inoculated into CAMH broth supplemented with serial concentrations of gamithromycin from 0.25× to 8× MICs (i.e. 0.03125 to 1 mg/L; MIC_broth_= 0.125 mg/L). After 3, 6, 9 and 24 h of incubation at 37 °C, serial dilution of each culture were plated on blood agar and incubated 18–24 h for the enumeration of viable bacterial counts. The detection limit for bacterial enumeration was 40 cfu/mL. Three independent replicates were performed for each curve.

### PAE and PA-SME determination

The PAE and PA-SME of gamithromycin against *P. multocida* CVCC 434 were determined using a spectrophotometric assay as previously described [16]. Briefly, *P. multocida* cells were exposed to gamithromycin at 1× and 4× MICs (i.e. 0.125 and 0.5 mg/L; MIC_broth_= 0.125 mg/L) for 1 h at 37 ℃. After centrifugation (3,000 g for 10 min) to remove drug, bacterial cells were resuspended in antibiotic-free broth or in broth containing gamithromycin concentrations of 0.1 to 0.3× MICs (i.e. 0.0125 and 0.0375 mg/L) for continuous measurement of absorbance at 600 nm. The optical density measurements were converted into bacterial counts (cfu/mL) according to a calibration curve. The calculations of PAEs and PA-SMEs have been described in detail elsewhere [21, 34].

### Animals and experimental design

Eighteen healthy New Zealand rabbits weighing 1.5–2.0 kg (Guangdong Medical Laboratory Animal Center, Guangzhou, China) were randomly divided into two equal groups, with nine rabbits in each group. Rabbits from both groups received a single IV (*via* the marginal vein of the left ear) or SC (*via* the loose skin over the neck) administration of gamithromycin, respectively, at a recommended dose of 6.0 mg/kg body weight that was previously approved for swine and cattle. In order to ensure the accuracy of the dosages for rabbits, gamithromycin injectable solution (Zactran, 150 mg/mL) was diluted 10-fold with 0.015 mol/L citrate buffer to a final concentration of 15 mg/mL before administration. The volume of injection was approximately 0.6 to 0.8 mL for each rabbit.

All rabbits were kept according to the institutional guidelines. Drinking water and antibiotic-free food were available *ad libitum* throughout the study. The animal study was approved by the Animal Research and Ethics Committee (IACUC) of South China Agricultural University (approval No. 2022E019).

### Sampling procedure and measurement of gamithromycin plasma concentrations

Blood samples (0.5 mL) were collected from the bilateral auricular veins of rabbits into heparinised vacutainers at 0.08, 0.25, 0.5, 1, 2, 4, 8, 12, 24, 48, 72 and 96 h after gamithromycin administrations. For IV administration, blood samples were collected solely from the contralateral (right) ear vein of the rabbits. The plasma was separated by centrifugation at 3000 rpm for 10 m*i*n and stored at -80 °C until assayed.

For drug extraction, a 50 µL aliquot of plasma sample was mixed with 50 µL of acetonitrile. After centrifugation at 10,000×g for 10 min, the supernatant was filtered through a 0.22 μm nylon syringe filter and collected into a vial for drug concentration determination. Gamithromycin concentrations in plasma were determined using a HPLC-MS/MS method (Agilent 1200 HPLC system; API 4000 triple quadrupole mass spectrometer) equipped with a short column (Waters Symmetry C18, 2.1 × 100 mm, 3.5 μm) as previously described [16, 18, 24], with minor modifications. The injection volume was 5 µL and temperature was maintained at 35 °C. Mobile phase consisted of (A) acetonitrile and (B) 0.1% formic acid in water using a gradient elution with a flow rate of 0.25 mL/min: 0–1.0 min (5–45% A), 1.0–3.0 min (45% A), 3.0–3.5 min (45 − 5% A) and 3.5–11 min (5% A). The mass conditions were as follows: ionspray voltage, 5500 V; curtain gas, 25 psi; nebulizer gas, 50 psi; source temperature, 550 °C. Multiple reaction monitoring (MRM) transitions from m/z [(M + 2 H) ^2+^] 389.7→83.1 and 389.7→115.2 were chosen for gamithromycin in the positive mode.

The coefficient of determination (R^2^) was above 0.99 in the linear concentration range of 0.001 to 0.5 mg/L. All plasma samples that had concentrations of > 0.5 mg/L were diluted proportionally with control plasma prior to extraction with acetonitrile. The limit of quantification was 0.001 mg/L. The mean extraction recoveries of gamithromycin from the five replicate assays (*n* = 5) ranged from 93.6 to 104% at the spiked drug concentrations of 0.01, 0.05 and 0.2 mg/L in plasma. The intraday coefficients of variation for replicate control samples within this range varied from 2.92 to 5.78%, and the interday coefficients of variation ranged from 3.96 to 6.32%.

### Pharmacokinetic analysis

The concentration-time data of gamithromycin in rabbits were submitted to a non-compartmental analysis. The calculations of all PK parameters were conducted using Phoenix WinNonlin software (Version 8.4, Certara, NJ, USA) and presented as mean ± SD. The bioavailability (F) of gamithromycin was calculated according to the standard equations as published previously [35].

### Protein binding of gamithromycin in rabbit plasma

The binding ratio of gamithromycin to rabbit plasma protein was determined using an equilibrium dialysis method as described [36–38]. Briefly, cellulose acetate dialysis bags with a molecular weight cut-off of 10 kDa (Shanghai Yuanye Biotechnology, China) were filled with fresh rabbit plasma and completely immersed in the dialysate (PBS solution, pH 7.4) at 37 °C for 24 h. The dialysate was spiked with gamithromycin resulting in a final concentration of 0.1, 1 or 10 mg/L. The HPLC-MS/MS method was used to quantify the drug concentration in the plasma and PBS dialysate. The fraction bound was calculated as ([plasma] - [dialysate])/[plasma] ×100% [39].

### Ex vivo antimicrobial activity and PK/PD relationship of gamithromycin against ***P. multocida***

The ability of gamithromycin to kill *P. multocida* (MIC) was assessed as previously described [20]. Ex vivo antimicrobial activity was determined separately using drug-containing plasma from individual rabbit at specified time points from 0 to 96 h after receiving subcutaneous dosing of gamithromycin at 6.0 mg/kg. Plasma samples were pre-filtered through a 0.22 μm filter to clear any possible bacterial contamination. *P. multocida* CVCC434 cultures at logarithmic growth phase were inoculated to each plasma sample, giving an initial inoculum of ~ 6 × 10^6^ cfu/mL. The mixtures were serially diluted and plated using a drop-plate technique to determine visible bacterial counts after 3, 6, 9 and 24 h of incubation. The limit of detection was 40 cfu/mL. For the ex vivo time-killing curves, gamithromycin concentrations in rabbit plasma were transformed to free drug concentrations by a mean percent protein binding of 25.7%. All curves are representative of three independent replicates.

The AUC_24h_/MIC ratio was chosen as the predictive PK/PD index to describe gamithromycin efficacy in the treatments of lung infections [15, 16]. The ex vivo antimicrobial activity (E) at a given gamithromycin concentration was expressed as the log_10_ change of bacterial count after 24 h of incubation. The correlation between antimicrobial activity and the AUC_24h_/MIC ratio was determined using the sigmoid E_max_ model in Phoenix WinNonlin 8.4 (Certara, NJ, USA) as follows: *E* = *E*_0_ + *E*_max_ × *C*^*N*^ / (*EC*_50_^*N*^ + *C*^*N*^), where *C* is the AUC_24h_/MIC ratio, *E*_0_ is the log_10_ change of bacterial count in drug-free control, *EC*_50_ is the AUC_24h_/MIC required to achieve 50% of the maximum effect (*E*_max_) and *N* is the Hill coefficient representing the slope of dose-response curve. The AUC_24h_/MIC ratios necessary for the bacteriostatic (E = 0), bactericidal (E = -3) and bacterial eradication (E = -4) effects were calculated using the E_max_ model against *P. multocida* infections in rabbit plasma.

### Electronic supplementary material

Below is the link to the electronic supplementary material.

Supplementary Material 1

## Data Availability

The datasets used and/or analyzed during the current study are available from the corresponding author on reasonable request.

## Abbreviations

PK/PD: pharmacokinetic/pharmacodynamic
PAE: post-antibiotic effect
PA-SME: post-antibiotic sub-inhibitory concentration effect
C_max_: peak concentration
T_1/2_: terminal half-life
AUC: area under the concentration-time curve
CAMH broth: cation-adjusted Mueller-Hinton broth
IV: intravenous
SC: subcutaneous
